# A Short Paper on the Nature of Inebriation, and the Means of Cure

**Published:** 1868-12

**Authors:** N. S. Davis

**Affiliations:** Chicago, Ill.


ARTICLE L.

### A SHORT PAPER ON THE NATURE OF INEBRIATION, AND THE MEANS OF CURE.

BY N. S. DAVIS, M.D., etc., Chicago, Ill.

[Prepared for the Western Association for the Advancement of Social Science, November 10th, 1868.]

There are no questions connected with the social interests of the human family more important than those which relate to the influence of intoxicating drinks. Whether we consider the vast pecuniary expenditure occasioned by their use; the impoverishment it helps to perpetuate among all classes; and the indirect effects upon the sanitary condition of individuals and communities; or whether we study their more direct influence over the moral, social, and physical condition of the consumer, we shall be overwhelmed with the magnitude of the subject, and astonished at the tenacity with which popular errors are perpetuated from generation to generation.

It is no part of our present purpose, however, to enter upon so broad a field of inquiry. But simply to offer a few suggestions in relation to the nature of *inebriation*, and the means that may be made available for its cure. For whatever differences of opinion may be entertained in regard to the propriety or impropriety of using intoxicating drinks, there is no escape from the fact, that such use tends so strongly to establish an appetite for more, that the number of inebriates among all classes of society is so great as to demand the serious attention of every good citizen.

It might appear to some superfluous to enter upon a serious inquiry into the *nature* of so familiar a condition as alcoholic inebriation. And yet we doubt whether there is any subject relating to the social interests of man, concerning which more mischievous errors exist in the popular mind. Perhaps four-fifths of the people at the present time regard inebriation or drunkenness simply as a vice, a crime, or moral delinquency, arising from the abuse of those fermented and distilled drinks which, properly used, are tonic, nutritive, and life-sustaining.

Hence, few things are more common, than to see a man or woman sipping wine, and at the same time expatiating upon the moral delinquency and social degradation of some neighbor, who, by long continuance of the same process, has become an habitual drunkard.

But is inebriation really a crime or a disease? Is it a state of moral turpitude, or a morbid condition of the physical organization, induced by the action of a material agent brought in contact with such organization through the blood? It may be a crime to drink an intoxicating draught for the purpose of inducing inebriation, or with a full knowledge that such will be the result; in the same sense, that it is a crime to deliberately expose ourselves to such atmospheric changes as will very certainly cause an attack of rheumatism or pneumonia, or otherwise impair our health and usefulness.

But that the appetite for alcoholic drinks and the state of inebriation are diseased conditions of certain organs or structures, is susceptible of clearest demonstration. To procure a full recognition of this fact by the community at large, is a step of paramount importance in preparing the way for the adoption of such measures as will either prevent or cure the disease. We cannot accomplish this object, however, without first establishing a correct knowledge of the nature and effects of alcohol, as the active ingredient in all the intoxicating drinks in common use, whether fermented or distilled. It is probable that a very large majority of the people, even at the present time, regard alcoholic drinks, when used with moderation, as tonic, nourishing, warming, and life-sustaining. To use the language of another, they are regarded as the "conservators of strength in manhood and as the milk of age." These popular notions are strengthened, on the one hand, by the direct exhilarating effect of alcohol on the nervous system, and on the other by certain theoretical dogmas promulgated by Liebig, Johnston, Hammond, and others, who have boldly proclaimed alcohol to be either respiratory or accessory food. This class of chemico-pathologists simply point to the fact that alcohol, in its chemical relations, belongs to the class of Hydrocarbons; and that these substances, out of the living body, are capable of undergoing combustion by uniting with an additional proportion of oxygen; and straightway jump to the conclusion that, when taken into the system, they actually enter into such combination with oxygen, and hence become respiratory food. And yet, we search all their writings in vain for the first item of proof that their mere theoretical deduction is correct.

A more recent modification of the theories emanating from this school of writers, makes alcohol, not respiratory, but *accessory food*. It having been clearly proved, by the experiments of Boker and others, that the presence of alcohol in the system lessened the atomic changes and secretions in such a way as to diminish the loss of weight, by diminishing the sum-total of eliminations in a given time, it was at once assumed that this diminution of atomic changes in the tissues of the body, was equivalent to just so much nutrition or addition of new matter through digestion and assimilation; and hence the alcohol was declared to be accessory or indirect food; a fallacy that will be exposed in another place.

We have thus stated fairly the theoretical doctrines of this class of men, because their names are continually quoted as authority throughout all departments of our literature.

Let us now see how these theoretical assumptions and popular notions are sustained by a wide range of experiments and carefully-observed facts.

*First.* Numerous chemical analyses of the blood and different tissues, made by different experimenters, show that when alcoholic drinks are taken, the alcohol enters the blood and permeates with it every part of the body.

This position is now acknowledged to be correct by all classes of observers.

*Second.* An equally reliable series of experiments have shown that the alcohol undergoes no chemical change in the system, but is eliminated through the excretory organs, more especially the lungs and kidneys, within a few hours after it is taken.

This position has long been disputed, but it was finally fully established by the results of the, well-devised and carefully-executed, experiments of Lallemand, Perrin, and Duroy.

*Third.* While in the blood and circulating through the system, the alcohol diminishes the sensibility of the brain and nervous system in the same manner as other anæsthetics, and also retards the active changes in all the tissues; and consequently diminishes the sum-total of eliminations or excretions in a given period of time.

The numerous and patient experimental investigations of Prout, Sandras, and Boucherdet, Boker, Hammond, and others, have removed all doubts in regard to the truth of this proposition.

*Fourth.* By diminishing the atomic changes in the tissues of the body and the sensibility of the nervous system, the alcohol by its presence also diminishes the temperature, the strength, and the power of endurance. That its presence in the system reduces the temperature, was first fully established by the results of a series of experiments performed by myself in 1850, some of which I repeated in 1867. These experiments consisted in testing the actual temperature of the body every half hour, with a delicately-graduated thermometer, for three hours after a moderate drink of alcoholic liquor. The tests were applied to both wine and whiskey.

These results are confirmed by the observations of Magnus and others in Europe. That the presence of alcohol directly diminishes the strength and power of endurance, is proved, not only by the foregoing scientific investigations, but also by a large number of carefully-observed facts in relation to the results of Labor, in civil and military life, and by the statistics of sickness and mortality.

Twelve or fifteen years ago an article was published in the *British and Foreign Medico-Chirurgical Review*, embracing a large amount of statistical information on this subject. At one place in England where a large amount of brick-making is carried on, and where the amount of each man's work, the number of days lost by sickness or otherwise, and the deaths, were made matters of record, the rules of the service allowed to every man a mug of beer at each meal.

But there were among the workmen quite a number who wholly abstained from the use of the beer, or any other intoxicating drink.

An examination of the record showed that the average amount of work done, per annum, by the beer-drinkers was a large percentage less than that done by those who wholly abstained, while the number of days lost by sickness was greater. The same relative results have been shown in reference to every other species of manual labor where records have been kept sufficient to afford a comparison. The article to which we have just alluded contained some interesting items from the reports of the Registrar-General of the British Army. At that time the soldiers of the army in the East Indies were allowed regular daily rations of whiskey; but there were some companies of pledged teetotalers, who faithfully declined the ration. On examining the details of the regular reports concerning sickness and mortality in that army, it was found that the ratio of sickness and mortality among the teetotalers was from five to ten per cent. less than for the rest of the army.

The history of our own army affords some facts of importance on this subject. Dr. Mann, a surgeon of excellent authority, who served with the army of the Revolution, says:—"At that period, during the Revolutionary War, when the army received no pay for their services, and possessed not the means to procure *spirits, it was healthy.* The 4th Massachusetts Regiment, at that eventful period of which I was surgeon, lost in three years, by *sickness*, not more than five or six men. It was a time when the army was destitute of money. During the winter of 1779–'80, there was only one occurrence of fever in the regiment, and that was a pneumonia of a mild form. It was observable in the last war, from December, 1814, to April, 1815, the soldiers at Plattsburg were not attacked with fevers as they had been the preceding winters. The troops during this period were not paid; a fortunate circumstance to the army, arising from the want of funds."

These may be said to be only negative results, but unhappily the history of the recent war for the suppression of rebellion furnishes some examples of a positive character. While General Cass was Secretary of War, several years since, the whiskey rations were discontinued in the United States Army, and coffee and sugar substituted. This has been the army regulation ever since. The Army of the Potomac, in the spring of 1862, was subjected to great hardships in labor, and exposed to the extremely wet and malarious region of the Chickahominy. There was consequently much sickness and suffering. Under these circumstances, the Commanding General issued an order on the 19th of May, allowing every officer and soldier one gill of whiskey per day; half to be served in the morning and half in the evening.

But the results were so manifestly injurious to the sanitary condition of the army, that in just thirty days the order was fully countermanded by the same General. Concerning this experiment in the Army of the Potomac, Dr. Frank H. Hamilton, one of the most eminent surgeons serving with that army, says:—"It is earnestly desired that no such experiment will ever be repeated in the armies of the United States. In our own mind, the conviction is established by the experience and observation of a life, that the regular routine employment of alcoholic stimulants by man in health is never, under any circumstances, useful. We make no exceptions in favor of cold, or heat, or rain, nor, indeed, in favor of old drinkers, when we consider them as soldiers."

For these and other facts bearing on this subject, see Dr. Hamilton's valuable work on Military Surgery, from page 70 to 75. Further facts of a striking and perfectly authentic character, bearing on this subject, may be found in one of the volumes of "Medical Inquiries and Observations, by Benjamin Rush, M.D.," in the chapter devoted to a comparison of the diseases prevalent in Philadelphia during ten years previous to the Revolutionary War, with those of a similar period after the close of that war. Indeed, it were easy to fill a volume with facts and statistics showing that in every relation of human life, the use of alcoholic drinks diminishes man's capacity to endure both mental and physical labor; increases his predisposition to disease; and shortens the average duration of life. And, although we have had our attention directed to this subject for thirty years, we have not found, either in the records of medicine or of general literature, a single statistical item calculated to prove the contrary. We have seen an abundance of *opinions* expressed of an adverse character. But *opinions* are not facts.

It is very common to hear that some sick or injured person has been kept up, or "kept alive," on brandy, or whiskey, or wine, for several weeks. But do those who make the assertion have any reliable means of knowing whether the sick person was actually kept alive by the alcoholic potion, or whether he lived in spite of it? The eminent Dr. Todd testified strongly to the sustaining and beneficial influence of alcoholic drinks in the low forms of fever, yet statistics show that in the London fever hospitals with which he was connected, the ratio of mortality increased *pari passu* with the increased use of alcoholic drinks as remedies. The able corps of medical attendants on the Bellevue and Emigrant Hospitals in New York also bore decided testimony to the utility of these liquors in the treatment of the same forms of fever, and used them largely. But the mortality was one in every five or six cases treated. The same fevers placed in tents with plenty of fresh air, and nourishment without a drop of alcoholic drinks in their treatment, gave a mortality of only one in seventeen.

The statistics of almost all epidemics show that those addicted to the use of intoxicating drinks are much more liable to be attacked than those who abstain; and of those who are attacked, a larger proportion die. If alcohol takes no part in nutrition, but passes through the blood disturbing the nervous sensibility and retarding the organic changes, some may be ready to ask why habitual drinkers of wine and beer grow fat and increase much in weight. We answer, that the presence of alcohol in the blood not only retards atomic changes in the tissues, but it diminishes the amount of oxygen taken in through the lungs. Consequently, the carbonaceous elements of the blood and tissues do not become oxidated as rapidly as when the alcohol is not present, and they accumulate in the form of *fat*. And this process is sometimes carried so far that the heart, liver, and kidneys undergo more or less fatty degeneration, constituting incurable forms of disease.

The increase of bulk and weight in these cases is not from an increase of natural nutrition, but a slow accumulation of hydro-carbonaceous material from retarded metamorphosis. The individual fattened under such influences, invariably diminishes in physical activity and power of endurance, in proportion to the increase of weight. Those who imagine that to diminish the waste of the tissues by diminishing the atomic changes, is equivalent to the actual assimilation and addition of new atoms, forget that all the phenomena of life in the physical organization, are the direct result of such atomic changes; and that whatever diminishes these actually diminishes physical life, and to stop them is to stop life.

If the reverse were true, we should only need enough of alcohol, or some other agent, to stop the changes in our tissues altogether, and we would live on indefinitely, without the trouble or expense of eating.

The simple logical deduction from all the carefully-observed facts, in relation to this subject, is, that alcoholic drinks, when taken into the human system, circulate in the blood as foreign agents; and until eliminated or cast out through the excretory organs, produce a temporary exhilaration of the mental faculties, accompanied by diminished sensibility, temperature, strength, and atomic changes. And when these effects are often induced, they result in a more or less permanent morbid or diseased condition of the organic actions, and especially of that portion of the nervous system connecting the stomach with the brain, constituting a morbid appetite or craving, which, in its demands for the accustomed exhilarant, often overpowers the most capacious and cultivated intellect and the most sacred pledges.

These deductions have a most important bearing on the question of how ought we to treat the inebriate? If the inebriate is the victim of a positive disease, induced by the action of an alluring and deceptive physical agent, alcohol, will any number of moral lessons addressed to his intellect, or any amount of denunciation hurled at his degradation and his vices, cure or reform him? Or, will his arrest, arraignment in a police court, and extortion of the few dollars he has left as a fine, eradicate the disease that is preying upon the most delicate part of his organization? Abundant experience throughout the civilized world answers these questions in the negative. The treatment demanded by the nature of inebriation, and the interests of humanity, is the same in kind as that awarded to the sick and the insane.

Instead of denunciations, fines, and imprisonments, the inebriate should be sent directly to an asylum, where he should receive thorough instruction in regard to the actual effects of alcoholic drinks on his physical as well as mental condition, in connection with such diet, medication, and employment, as a specially qualified superintendent should deem adapted to his case. The establishment of Washingtonian Homes and Inebriate Asylums in Boston, Chicago, Binghamton, New York, and a few other places, is only the initiatory step in this great work. These institutions are adapted to the reception and treatment of such inebriates as voluntarily resort to them. But to reach the great mass of those suffering from the disease of inebriation, requires a thorough change in our jurisprudence.

The law must recognize the important fact that inebriation is temporary insanity, caused by the morbid effect of a physical agent on the brain and nervous system. Instead of arrests, petty fines, and temporary imprisonments in police stations, Bridewell, etc., ending only in a further demoralization and speedy return to the dram-shop, the law must provide well-appointed asylums, in which the victims of alcoholic disease can be legally placed, until, by the combined influence of correct instruction, abstinence, productive labor, and proper medication, the disease and morbid appetite are effectually removed. Such a change in the management of drunkenness would speedily work other changes of vital importance to society.

Alcoholic drinks, becoming directly associated with the idea of disease and mental alienation, in the public mind, would speedily come to be universally regarded in their true light as debilitating to body and mind, instead of tonic and life-sustaining. This would necessarily be accompanied by a corresponding change in the language of the physician at the family fireside, and in the phraseology adopted in the press and the current literature of the day. Such a change would do more to discourage dram-drinking, and all its direful consequences, than all other measures combined. We hope, therefore, that the members of this Association will give to this subject all that thought and patient investigation which its importance demands.

